# The Psychology of Fair Assessment: Mindfulness, Mental Health, and Grading Bias Among Senior High School Teachers in Ghana

**DOI:** 10.1002/brb3.71779

**Published:** 2026-09-17

**Authors:** Simon Ntumi

**Affiliations:** ^1^ Department of Educational Foundations University of Education, Winneba (UEW) Winneba Ghana

**Keywords:** assessment fairness, grading bias, job satisfaction, mediation, mental health, mindfulness, moderation, psychological distress

## Abstract

**Background:**

In recent years, teacher well‐being and fairness in assessment have emerged as critical issues in higher education, particularly in contexts of rising stress and burnout. This study employed a correlational research design to investigate the relationships among mindfulness, psychological distress, mental health, and assessment fairness among senior high school teachers in Ghana.

**Method:**

A sample of 1024 teachers from public and private institutions was recruited using stratified random sampling to ensure representation across key demographics. Adopted validated psychometric tools assessed mindfulness (Five Facet Mindfulness Questionnaire [FFMQ]), psychological distress (Kessler Psychological Distress Scale (10 items) [K10]), mental health status (General Health Questionnaire (12 items) [GHQ‐12]), emotional exhaustion (Maslach Burnout Inventory‐Emotional Exhaustion subscale [MBI‐EE]), job satisfaction survey (JSS), and perceptions of grading bias and fairness. Data were collected via online surveys, enabling wide geographical reach and participant anonymity.

**Result:**

The results revealed that mindfulness was positively associated with mental health, job satisfaction, and perceptions of assessment fairness, and negatively correlated with psychological distress, emotional exhaustion, and grading bias tendencies. Multiple regression analysis showed that mindfulness (*β* = 0.30, *p* < 0.001**), mental health (*β* = 0.22, *p* = 0.003**), and job satisfaction (*β* = 0.25, *p* = 0.001**) significantly associated assessment fairness perceptions. Conversely, psychological distress (*β* = −0.24, *p* = 0.001**), emotional exhaustion (*β* = −0.21, *p* = 0.002**), and grading bias tendency (*β* = −0.27, *p* = 0.001**) were negative associators. Moderation analysis indicated that mindfulness buffered the detrimental effect of psychological distress on fairness perceptions (interaction *β* = 0.21, *p* = 0.003**). Mediation analysis further showed that mental health partially mediated the mindfulness–assessment fairness relationship (indirect *β* = 0.13, 95% confidence interval [CI] [0.08, 0.21]), suggesting mindfulness fosters fairness perceptions partly by enhancing mental well‐being.

**Conclusion:**

The findings highlight key psychological pathways through which mindfulness supports equitable assessment practices, emphasizing the importance of mindfulness‐based interventions and mental health support to promote fairness, reduce bias, and improve teachers’ well‐being in senior high school education settings.

## Graphical abstract

Mindfulness among Ghanaian university educators enhances mental health and job satisfaction while reducing distress, emotional exhaustion, and grading bias. By buffering stress and partially operating through improved mental well‐being, mindfulness promotes fairer and more equitable assessment practices in higher education.

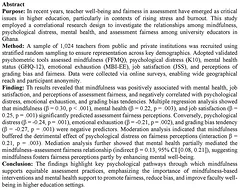

**Abbreviations**
AF: assessment fairness
EE: emotional exhaustion
FFMQ: Five Facet Mindfulness Questionnaire
GB: grading bias (or grading bias tendency)
GES: Ghana Education Service
GHQ‐12: General Health Questionnaire (12 items)
JS: job satisfaction
JSS: Job Satisfaction Scale
K10: Kessler Psychological Distress Scale (10 items)
MBI‐EE: Maslach Burnout Inventory‐Emotional Exhaustion subscale
MBSR: mindfulness‐based stress reduction
MH: mental health
PD: psychological distress

## 1 Introduction

In the dynamic and often high‐pressure environment of modern education, teachers are increasingly exposed to elevated levels of psychological distress (PD), emotional exhaustion (EE), and professional burnout (Maslach and Leiter 2016; Maslach et al. 2001; Skaalvik and Skaalvik 2017). The multifaceted demands placed on educators including curriculum delivery, administrative responsibilities, classroom management, student engagement, and parental communication often result in chronic stress that can compromise both personal and professional well‐being (Chang 2019; Mackey 2022; Sokal et al. 2020; Skaalvik and Skaalvik 2017). These pressures have been further exacerbated by educational reforms, high‐stakes testing, large class sizes, and, in some contexts, the residual effects of the COVID‐19 pandemic, which has intensified workload and emotional labor (Rafferty 2025; Ntumi et al. 2026d; Sokal et al. 2020). As a result, many teachers report symptoms of anxiety, sleep disturbances, feelings of depersonalization, and a diminished sense of accomplishment, all of which are hallmark indicators of burnout (Maslach et al. 1996; Maslach et al. 2001; Chang 2019). The mental health (MH) of educators is not only central to their personal well‐being but also significantly influences pedagogical effectiveness, classroom climate, and professional judgment, including assessment practices (Quinn 2020; Ntumi et al. 2026a). Teachers experiencing high levels of distress may struggle to maintain emotional regulation, deliver high‐quality instruction, or engage meaningfully with students. Importantly, stress‐related impairments in attention, decision‐making, and empathy may also affect how teachers perceive and evaluate student performance. For example, emotionally depleted teachers may rely more on cognitive shortcuts, stereotypes, or rigid heuristics when grading, potentially introducing unintentional bias and compromising the fairness of assessments (Okonofua and Eberhardt 2015). This is especially critical given that grading is not merely a technical act but also an interpretive process influenced by the grader's emotional and cognitive state (Brookhart 2011; Ntumi and Bulala 2025).

In recent years, the role of teacher well‐being in shaping educational equity and fairness has received growing scholarly attention. Researchers are beginning to connect the dots between teacher MH and systemic issues such as student achievement gaps, discipline disparities, and biased academic evaluations (Ntumi et al. 2026c; Quinn 2020; Nur'aeni and Patry 2024). In this context, promoting teacher well‐being is no longer viewed solely as a matter of self‐care or occupational health but also as a strategic lever for educational justice. The intersection of educator stress and assessment fairness (AF) therefore represents a critical area for intervention and innovation, particularly as schools seek holistic approaches to fostering supportive and equitable learning environments. One promising approach to enhancing teacher well‐being and reducing PD is mindfulness‐based stress reduction (MBSR), an evidence‐based program that incorporates mindfulness meditation, body awareness, and gentle yoga to cultivate greater awareness of the present moment and reduce reactivity to stress (Brown and Ryan 2003; Nur'aeni and Patry 2024; Roeser et al. 2013). Grounded in ancient contemplative traditions and contemporary clinical psychology, MBSR teaches participants to observe their thoughts and emotions with nonjudgmental awareness, thereby fostering greater emotional regulation and psychological flexibility. Originally developed for patients coping with chronic pain and stress‐related illnesses, MBSR has since been successfully adapted for a variety of populations and settings, including healthcare, corporate environments, and more recently, education (Emerson et al. 2017; Roeser et al. 2013).

In school contexts, MBSR has shown significant promise in mitigating teacher stress, reducing burnout, and improving indicators of mental resilience such as self‐compassion, attentional control, and emotional balance (Aeschbach et al. 2022; Hanif et al. 2025; Jackman et al. 2025; Rombouts et al. 2025). Teachers who undergo mindfulness training report not only improved mood and well‐being but also enhanced relational skills, such as greater patience, empathy, and attunement to students’ needs. These improvements can have downstream effects on instructional quality and classroom dynamics, potentially reducing conflict and promoting a more inclusive learning atmosphere (Phan et al. 2025; Roeser et al. 2013). Importantly, mindfulness practices may also influence how teachers approach complex tasks like grading, by fostering a more reflective, less reactive mindset. As such, the potential impact of MBSR extends beyond individual health to the ethical dimensions of teaching, particularly in promoting fairness, consistency, and reduced bias in evaluative practices. Given the increasing prevalence of mindfulness initiatives in educational settings, there is a growing need for rigorous, empirical investigation into their broader implications (Emerson et al. 2017; Rombouts et al. 2025). Specifically, research is needed to examine whether and how MBSR might affect not only teacher MH but also core professional practices such as assessment. Exploring the link between mindfulness, emotional regulation, and grading decisions may provide valuable insights into how interventions targeting teacher well‐being can contribute to greater educational equity and integrity (Quinn 2020).

AF, broadly defined as the unbiased and consistent application of evaluation criteria across students, is a cornerstone of educational integrity and a fundamental requirement for equitable learning environments (Black and Wiliam 2019; Quinn 2020). Fair assessment ensures that students are judged based on clearly articulated standards and demonstrated competencies rather than on extraneous factors such as personal characteristics, background, or teacher dispositions. It underpins students’ trust in the educational system and contributes to their motivation, sense of belonging, and academic self‐concept (Brookhart 2011; Quinn 2020). However, maintaining fairness in assessment is a cognitively and emotionally demanding task, particularly in high‐stakes, high‐pressure educational contexts where teachers are expected to make numerous evaluative decisions under conditions of limited time and elevated stress. Empirical research has consistently shown that teacher bias whether implicit, affective, or stress‐induced can significantly skew grading practices, often in ways that disadvantage marginalized or underrepresented student populations (Ntumi et al. 2026a; Okonofua and Eberhardt 2015). Implicit biases, rooted in societal stereotypes and unconscious associations, may lead teachers to systematically overestimate or underestimate students’ abilities based on race, gender, socioeconomic status, or linguistic background (Quinn 2020). For instance, studies have found that students from minority ethnic groups are more likely to receive harsher evaluations even when their performance is equivalent to their peers (Okonofua and Eberhardt 2015). Moreover, subjective elements of assessment such as essays, participation, and behavior‐based evaluations are particularly vulnerable to these biases.

Compounding this issue is the role of teachers’ emotional and psychological states in shaping their professional judgments. Emotional conditions such as fatigue, anxiety, burnout, or irritability common in the teaching profession have been empirically linked to impaired cognitive functioning and a reduced capacity for objective, nuanced judgment (Chang 2019; Maslach et al. 2001). Under such circumstances, individuals are more likely to rely on cognitive shortcuts, such as heuristics or stereotypes, to simplify complex decision‐making tasks (Sinclair and Kunda 2000). This suggests that stressed teachers may unknowingly allow emotional reactivity to distort their assessment practices, leading to inconsistencies in grading and reinforcing systemic inequities. Given these dynamics, the psychological and emotional regulation of educators is emerging as a key variable in safeguarding AF. Mindfulness, with its emphasis on present‐moment awareness, emotional self‐regulation, and nonjudgmental observation, offers a promising mechanism for interrupting the automatic activation of biases and fostering more deliberate, equitable decision‐making processes (Baer et al. 2006; Baer et al. 2008). By cultivating cognitive clarity and emotional stability, mindfulness may help teachers recognize and manage the internal states that can compromise their objectivity. This has important implications not only for teacher well‐being but also for the ethical foundations of teaching practice.

Consequently, exploring the role of mindfulness in moderating the emotional states that contribute to bias such as anxiety, stress, and irritability provides an important and timely avenue for promoting AF in schools (Munawar et al. 2025; Pascoe et al. 2017). If mindfulness‐based interventions such as MBSR can attenuate the psychological precursors of biased judgment, they may serve a dual function: enhancing teacher well‐being while simultaneously improving the validity and fairness of grading practices. This intersection of MH, mindfulness, and ethical assessment represents a critical, yet underexplored, domain in educational research, one that warrants further empirical attention in the pursuit of both educator flourishing and systemic equity. This study therefore sought to investigate the dual impact of MBSR on (1) teacher PD and MH and (2) the fairness of their grading practices. Drawing upon interdisciplinary theories from educational psychology, contemplative science, and assessment theory, this research addresses a critical gap at the intersection of teacher well‐being and educational justice. By examining whether participation in an MBSR program leads to measurable improvements in MH and reduces cognitive–emotional factors that may contribute to biased assessments, this study contributes to a growing body of literature advocating for mindfulness as both a personal wellness and pedagogical equity intervention.

### 1.1 MBSR Model and Its Applicability to the Study

MBSR is a well‐established, evidence‐based intervention program developed by Jon Kabat‐Zinn at the University of Massachusetts Medical Center. Originally designed to help individuals cope with chronic pain and stress‐related disorders, MBSR has since been widely adapted across various clinical and nonclinical settings, including education. The core of the MBSR program lies in cultivating mindfulness, which Kabat‐Zinn (1994) and Brown and Ryan (2003) define as the intentional, nonjudgmental awareness of the present moment. The program typically spans 8 weeks and incorporates a range of formal mindfulness practices such as sitting meditation, body scanning, and gentle yoga, alongside informal practices that encourage participants to bring mindful awareness into everyday activities. Additionally, psychoeducation and group discussions are integral components that support participants in understanding the mind–body relationship and developing skills to manage stress more effectively. The comprehensive nature of MBSR equips individuals with tools to observe their thoughts, emotions, and bodily sensations with curiosity and acceptance rather than judgment or avoidance.

The therapeutic effects of MBSR are well documented and include several interrelated mechanisms. One of the key benefits is enhanced emotional regulation, which refers to the improved ability to recognize, tolerate, and modulate distressing emotions without being overwhelmed or acting impulsively (Roeser et al. 2013). This is particularly relevant in high‐stress professions such as teaching, where emotional challenges can directly affect professional judgment and interactions. Furthermore, MBSR fosters cognitive flexibility by helping individuals disengage from automatic and habitual thought patterns, promoting more adaptive and deliberate responses to complex or stressful situations (Roeser et al. 2013; Baer et al. 2008). Another important mechanism is the reduction of rumination and worry, which are often contributors to anxiety and depressive symptoms; mindfulness anchors awareness in the present moment, thereby interrupting cycles of negative repetitive thinking (Hölzel et al. 2011; Gu et al. 2015). On a physiological level, mindfulness practice has been linked to reduced cortisol levels and enhanced autonomic nervous system functioning, which together alleviate the detrimental health effects of chronic stress (Roeser et al. 2013; Pascoe et al. 2017). Collectively, these mechanisms contribute to improved psychological well‐being and resilience.

The applicability of the MBSR model to the current study, which examines the impact of mindfulness on teacher well‐being and AF, is both timely and compelling. Teaching is a profession characterized by frequent exposure to emotional and cognitive stressors that can lead to burnout, anxiety, and EE (Roeser et al. 2013; Emerson et al. 2017; Jackman et al. 2025; Hanif et al. 2025). These psychological challenges not only impair teachers’ health but can also compromise their professional effectiveness, including critical decision‐making tasks like grading and assessment. MBSR directly addresses these issues by equipping teachers with mindfulness skills that reduce stress and improve their capacity to regulate emotions. Enhanced emotional regulation, in turn, supports teachers in maintaining composure and clarity in evaluative tasks, mitigating the influence of transient emotional states such as fatigue or irritability that can bias assessment judgments. This is crucial because grading requires objective and consistent application of standards, which can be compromised by emotional reactivity or cognitive overload. Moreover, MBSR's focus on cultivating present‐moment awareness and nonjudgmental observation enables teachers to identify when implicit biases or stress‐induced heuristics might be influencing their grading practices. Implicit bias, often unconscious and automatic, is a significant concern in educational assessment, with research showing that it can disproportionately disadvantage marginalized students (Okonofua and Eberhardt 2015). Under stressful conditions, cognitive shortcuts such as stereotyping become more prevalent as individuals seek to reduce cognitive load (Brookhart 2011; Maslach et al. 2001; Tang et al. 2015). Mindfulness practice disrupts these automatic processes by fostering meta‐cognitive awareness—the ability to observe one's own thoughts and feelings without immediate reaction. This capacity to “step back” from automatic responses is critical in promoting more equitable and fair grading decisions, as it allows teachers to pause and reflect rather than respond impulsively to stress or unconscious biases.

In addition to improving emotional and cognitive regulation, MBSR offers long‐term benefits for teacher well‐being and professional sustainability. Mindfulness fosters resilience and self‐care habits that help teachers manage ongoing stress and prevent burnout (Roeser et al. 2013; Emerson et al. 2017; Brown et al. 2007). This sustained well‐being not only supports individual teachers’ health but also contributes positively to the broader school environment, promoting a more supportive and less reactive culture. Mindful teachers are more likely to exhibit patience, empathy, and compassion qualities that enhance interpersonal relationships with students and colleagues and encourage ethical educational practices (Tang et al. 2015; Hölzel et al. 2011; Nur'aeni and Patry 2024). These qualities underpin the moral and ethical dimensions of teaching, including the commitment to fairness and equity in student assessment. Empirical evidence supports the relevance of MBSR for addressing the dual goals of teacher well‐being and AF. Roeser et al. (2013) demonstrated that teachers who participated in MBSR showed significant reductions in occupational stress and improvements in attention regulation, which translated into better classroom management and professional effectiveness. Emerson et al. (2017) reported that mindfulness‐based interventions effectively reduced teacher burnout and enhanced emotional well‐being, with secondary benefits for student–teacher relationships and classroom climate. Furthermore, Jackman et al. (2025) found that mindfulness training improved executive functioning and emotional regulation in educators, critical faculties that underlie objective and fair evaluation practices. These findings collectively suggest that MBSR can serve as a strategic intervention that not only promotes teacher MH but also upholds the integrity and fairness of assessment practices. In effect, the MBSR model offers a comprehensive framework that aligns closely with the aims of this study. By fostering emotional regulation, cognitive flexibility, and present‐moment awareness, MBSR equips teachers to manage the psychological stressors inherent in their profession and reduces the likelihood that these stressors will bias their assessment decisions. Integrating MBSR into teacher professional development is therefore a promising pathway to enhancing both individual well‐being and educational equity. The model's well‐documented efficacy and broad applicability make it an ideal theoretical and practical foundation for investigating how mindfulness interventions can impact teacher MH and promote fairness in grading practices, ultimately contributing to healthier educators and more just educational systems.

#### 1.1.1 Theoretical Foundation of the Hypotheses

The hypotheses are grounded in the Job Demands–Resources (JD–R) theory, which proposes that employee well‐being and work outcomes are influenced by the interaction between job demands and available resources. Job demands such as PD and EE can create strain, whereas resources help employees cope with demands and maintain positive functioning (Bakker and Demerouti 2024). In this study, mindfulness is conceptualized as a personal resource that may help teachers regulate their responses to psychological demands, while MH and job satisfaction (JS) represent positive aspects of teacher well‐being. The theory supports the expectation that mindfulness will be associated with lower PD and EE and better MH and JS. It also suggests that teachers with greater personal resources and better well‐being may be more capable of maintaining fair and consistent assessment practices. Consequently, mindfulness, MH, and JS are expected to positively correlate AF, whereas PD, EE, and grading bias (GB) tendency are expected to negatively correlate it. The JD–R theory further supports the moderating role of mindfulness, as personal resources can buffer the negative effects of demands (Bakker and Demerouti 2024). Thus, mindfulness is expected to weaken the negative relationship between PD and AF. Finally, the theory supports the mediating role of MH, whereby mindfulness may enhance MH, which subsequently contributes to more positive perceptions of AF (Grover et al. 2018). Overall, the JD–R framework provides the theoretical basis for the study's hypotheses by explaining how psychological demands and personal resources may jointly influence teachers’ well‐being and perceptions of AF (Kwon and Kim 2020; Rattrie et al. 2020).

#### 1.1.2 Hypotheses

**Hypothesis 1**. *Mindfulness is significantly associated with teacher well‐being, including PD, EE, JS, and MH status among senior high school teachers*.
**Hypothesis 2**. *Mindfulness, teacher well‐being, and GB tendency significantly correlate teachers’ perceptions of AF*.
**Hypothesis 3**. *Mindfulness significantly moderates the relationship between PD and teachers’ perceptions of AF*.
**Hypothesis 4**. *MH status significantly mediates the relationship between mindfulness and teachers’ perceptions of AF*.

## 2 Methods

### 2.1 Study Design

The study employed a correlational research design to investigate the relationships among mindfulness, PD, MH, and AF among senior high school teachers in Ghana. A correlational design was deemed appropriate because the primary aim was to determine the direction and strength of associations between variables specifically, to assess how levels of mindfulness, as cultivated through MBSR, relate to teacher well‐being and perceived fairness in grading practices. Correlational research does not involve manipulation of variables but rather observes naturally occurring relationships, making it ideal for psychological and educational research settings (Gay et al. 2012; Cohen et al. 2018). The decision to utilize a correlational framework also aligned with the exploratory nature of the study, which sought to establish empirical links that could inform future experimental or longitudinal research. This approach allowed for the examination of multiple interrelated constructs of mindfulness, PD, MH status, and GB within a real‐world teaching context. Furthermore, since causality was not the focus, but rather the pattern and strength of statistical associations, this design offered the flexibility and analytical robustness necessary for such multifactorial relationships. Given the widespread geographical distribution of the target population and the need for cost‐effective, time‐efficient data collection, an online survey approach was adopted. Online surveys have become increasingly valuable in educational research due to their ability to reach diverse populations rapidly while minimizing logistical barriers such as travel costs and manual data entry errors. The anonymity afforded by online responses may also have encouraged greater honesty among participants, particularly when answering items related to psychological well‐being and assessment bias, which can be sensitive topics in professional settings.

### 2.2 Population and Sampling Procedure

The target population for this study consisted of senior high school teachers employed in public and private institutions across all 16 administrative regions of Ghana. This group was chosen because they operate in high‐stakes academic environments where the psychological demands of the profession and the implications of assessment decisions are particularly pronounced. These teachers are also more likely to be engaged in formal grading processes that influence student progression, making them a relevant population for examining both well‐being and AF. To improve transparency regarding sampling and representativeness, the study used a stratified sampling framework based on geographical zone (Northern, Middle, and Coastal), school type (public and private), and school location (urban and rural), using senior high school lists obtained from the Ghana Education Service (GES). Schools were selected within the identified strata, after which eligible teachers in the selected schools were invited to participate. Teachers were required to be actively teaching in a senior high school and to have at least 1 year of teaching experience. Because recruitment also involved teacher email networks, WhatsApp and Telegram groups, union communication platforms, and regional education portals, the final procedure incorporated an element of voluntary/open‐link participation rather than a strictly controlled within‐school random selection of individual teachers. Consequently, the study does not claim that the final sample is fully nationally representative. Rather, the 1024 valid responses provide broad coverage of teachers across Ghana's 16 administrative regions and diverse school contexts, but findings should be generalized cautiously to the wider population of senior high school teachers.

The recruitment approach and voluntary nature of participation are acknowledged as potential sources of self‐selection bias. The recruitment message included the purpose of the study, ethical assurances, and a direct link to the online survey. Participation was voluntary, and teachers were required to meet eligibility criteria: They had to be actively teaching in a senior high school and possess at least 1 year of teaching experience to ensure they had engaged in formal assessment practices. In total, 1024 valid responses were received and included in the final analysis. This sample size exceeded the minimum threshold recommended for correlational studies involving multiple variables, which typically require between 300 and 500 participants to ensure robust statistical power and generalizability. The large sample allowed for disaggregated analysis by region, gender, and years of teaching experience, providing a nuanced understanding of the factors influencing teacher well‐being and grading practices in the Ghanaian context.

### 2.3 Instrument and Measures

Data for this study were collected using a structured online questionnaire hosted on Google Forms, designed to capture quantitative information on the core constructs of the research: mindfulness, PD, MH status, EE, JS, and AF. The questionnaire was organized into seven comprehensive sections: (1) demographic data, (2) mindfulness, (3) PD, (4) MH status, (5) EE, (6) JS, and (7) perceived fairness in assessment practices. The mindfulness construct was measured using the Five Facet Mindfulness Questionnaire (FFMQ) developed by Baer et al. (2008). This instrument evaluates mindfulness across five theoretically grounded dimensions: observing, describing, acting with awareness, non‐judging of inner experience, and non‐reactivity to inner experience. Each item was rated on a five‐point Likert scale ranging from 1 (“Never or very rarely true”) to 5 (“Very often or always true”). The FFMQ has demonstrated high construct validity and reliability in educational and occupational populations, making it well‐suited for use among teachers.

PD was assessed using the Kessler et al. (2002) PD Scale (K10), a 10‐item self‐report measure widely used in epidemiological and MH research to screen for nonspecific symptoms of anxiety and depression. Respondents indicated the frequency of specific feelings (e.g., nervousness, hopelessness, restlessness) over the past 30 days using a five‐point Likert scale ranging from 1 (“None of the time”) to 5 (“All of the time”). The K10 has been validated across diverse cultural contexts, including Sub‐Saharan Africa, and is sensitive to detecting PD in nonclinical populations. The MH status of teachers was evaluated using the General Health Questionnaire (GHQ‐12), a widely recognized instrument developed by Jackson (2007) to screen for general psychological well‐being and potential psychiatric morbidity. The GHQ‐12 focuses on the respondent's ability to carry out normal functions and the appearance of new and distressing experiences. Items were rated on a four‐point scale, with higher scores indicating poorer MH. The GHQ‐12 has been extensively used in educational settings and is known for its brevity and high predictive validity (Jackson 2007).

EE was measured using the Maslach Burnout Inventory‐EE subscale (MBI‐EE) originally developed by Maslach et al. (1996) and De Beer and Bianchi. This subscale includes items assessing feelings of being emotionally overextended and exhausted by one's work. Respondents rated their experiences on a seven‐point frequency scale ranging from 0 (“Never”) to 6 (“Every day”). The MBI‐EE is the most widely used instrument for assessing burnout in occupational groups, with strong psychometric properties in education professionals. JS was assessed using the JS Survey (JSS) developed by Spector (1997). The JSS comprises items that measure various facets of JS including pay, promotion, supervision, benefits, contingent rewards, operating conditions, coworkers, nature of work, and communication. Respondents rated their agreement on a six‐point Likert scale from 1 (“Disagree very much”) to 6 (“Agree very much”). The JSS is widely validated in educational contexts and provides a reliable multidimensional assessment of satisfaction with work.

GB tendency was measured using an eight‐item researcher‐developed GB Tendency Scale (GBTS), developed from literature on assessment bias, teacher judgment, teacher expectations, and grading practices and contextualized to Ghanaian senior high schools. The scale assessed differential treatment of students, the influence of teacher expectations, leniency or severity in grading, and the influence of nonacademic factors on grading decisions. Items were rated on a five‐point Likert scale ranging from 1 (strongly disagree) to 5 (strongly agree), with higher scores indicating greater grading‐bias tendency. Illustrative items included “I sometimes award different grades to students who perform at the same academic level because of my expectations of them” and “Non‐academic characteristics of students can influence the grades I assign.” The items were reviewed by experts in educational measurement and psychology for relevance, clarity, and content representativeness and were pilot‐tested with 30 senior high school teachers. The scale was conceptualized as a unidimensional measure of overall GB tendency, with the items capturing related manifestations of the construct. The GBTS demonstrated satisfactory internal consistency, with a Cronbach's *α* = 0.84. The reported coefficient was based on the pilot‐study responses. The expert review provided preliminary evidence of content validity, while the pilot testing provided evidence of item clarity and suitability for the study context.

Perceptions of AF were measured using a 12‐item adapted scale developed based on theoretical principles. This scale assessed dimensions such as consistency in grading criteria, impartiality of judgment, equity across student demographics, and transparency of assessment processes. Teachers responded using a five‐point Likert scale from 1 (“Strongly Disagree”) to 5 (“Strongly Agree”). The scale was contextually adapted for the Ghanaian senior high school environment to ensure cultural and occupational relevance. Prior to full deployment, the questionnaire was piloted with a sample of 30 senior high school teachers in the Greater Accra Region to assess clarity, internal consistency, and item relevance. Feedback from the pilot led to minor revisions in wording to enhance comprehension. Reliability analysis yielded Cronbach's alpha values ranging from 0.81 to 0.91 across the various subscales, indicating strong internal consistency and reliability. The final instrument demonstrated a high degree of psychometric robustness suitable for large‐scale correlational analysis.

### 2.4 Data Collection Procedure

Data collection was conducted over a 6‐week period between June and July 2025, following ethical approval from my University's Research Ethics Committee. To ensure comprehensive participation, a multipronged recruitment strategy was employed. A survey invitation link embedded within a brief description of the study's objectives, participant eligibility criteria, and ethical safeguards was electronically disseminated via platforms frequently used by teachers in Ghana, including official WhatsApp groups, Telegram channels, teacher union mailing lists, and regional education directorate portals. Participation was strictly voluntary, and respondents were provided with detailed informed consent information at the beginning of the survey. They were assured of anonymity and confidentiality, with no identifiable information collected. Only teachers who met the inclusion criteria, namely, being currently employed as a senior high school teacher in Ghana and having at least 1 year of teaching experience were eligible to complete the questionnaire. This ensured that all participants had relevant experience with grading practices and were capable of responding meaningfully to questions on AF. To maintain data integrity, the survey did not use IP‐address or device‐based blocking because no identifying information was collected and the survey was designed to remain anonymous. Instead, duplicate and potentially invalid responses were identified during data cleaning by examining repeated or highly similar response patterns, demographic information, completion times, and response completeness. Responses with substantial missing data, duplicate patterns, or indications of invalid responding were excluded. Only responses with at least 80% of the questionnaire completed and meeting the established data‐quality criteria were retained for analysis. Following this screening process, 1024 valid responses were obtained.

### 2.5 Data Analysis

Upon completion of data collection, all responses were exported from Google Forms into IBM SPSS Statistics version 27 and AMOS for comprehensive statistical analysis. The initial stage of the analysis involved data cleaning, during which responses were screened for completeness, duplicate entries, and outliers. Only responses with at least 80% completion were retained, and any inconsistencies were resolved prior to coding. Descriptive statistics were first computed to summarize demographic variables such as age, gender, teaching experience, regional distribution, and type of school (public vs. private). Measures of central tendency (mean, median) and dispersion (standard deviation, range) were used to describe the distribution of scores on the mindfulness, PD, MH status, and AF scales. To examine the relationships among key psychological constructs, the study employed Pearson product‐moment correlation coefficient analyses. These correlations allowed for the identification of statistically significant associations between mindfulness (as measured by the FFMQ), PD (K10), MH status (GHQ‐12), and perceived fairness in assessment. Correlation coefficients were interpreted based on Cohen's guidelines, with *r* values of 0.10, 0.30, and 0.50 indicating small, medium, and large effects, respectively.

Subsequently, multiple regression analyses were conducted to determine the extent to which mindfulness and MH variables significantly associated perceptions of AF. These regression models controlled for relevant demographic covariates such as years of teaching experience and school location (urban vs. rural). The aim was to assess whether teachers who demonstrated higher mindfulness traits also exhibited greater psychological well‐being and fairness in their grading practices. In addition to these traditional inferential techniques, the study employed structural equation modeling (SEM) using AMOS software to test a hypothesized model grounded in the theoretical assumptions of the MBSR framework. SEM enabled the simultaneous estimation of multiple dependent relationships between latent constructs, providing a holistic test of the conceptual framework. Key fit indices were used to evaluate the adequacy of the model, including the comparative fit index (CFI), Tucker–Lewis Index (TLI), root mean square error of approximation (RMSEA), and the Chi‐square to degrees of freedom ratio (*χ* ^2^/df). Following Nunnally and Bernstein (1994) recommendations, a CFI and TLI of ≥ 0.90, RMSEA ≤ 0.08, and a *χ* ^2^/df ratio of < 3 were considered indicative of good model fit. Modification indices were also consulted to determine if improvements in the model could be achieved by adding or removing specific paths. This comprehensive approach allowed for rigorous testing of the mediating and associative roles of mindfulness in the relationship between teacher stress and AF. All statistical tests were conducted at a 95% confidence level, with a significance threshold set at *p* < 0.05. The findings were interpreted in line with both statistical significance and practical relevance, ensuring meaningful implications for educational policy and teacher training.

### 2.6 Ethical Considerations

The study adhered strictly to ethical standards for research involving human subjects. Prior to the commencement of data collection, ethical clearance was obtained from the Institutional Review Board (IRB) of my University. The IRB reviewed the study protocol, instruments, and consent procedures to ensure that participants’ rights, safety, and well‐being were adequately protected. Participants were presented with a comprehensive informed consent form at the beginning of the online survey, detailing the purpose of the study, the voluntary nature of participation, expected duration, and potential risks and benefits. Consent was obtained electronically by requiring participants to check a box indicating their agreement before proceeding to the questionnaire. Respondents were also informed of their right to withdraw at any stage without facing any negative consequences. In alignment with the ethical principles outlined in the National Commission for the Protection of Human Subjects of Biomedical and Behavioral Research (1979) respect for persons, beneficence, and justice the research ensured that participation was equitable and nondiscriminatory. No teacher was coerced or incentivized in a way that might unduly influence their decision to participate. Furthermore, efforts were made to ensure that the sample included voices from diverse regions and school types to uphold the principle of justice. To safeguard confidentiality and data privacy, no personally identifying information was collected. All responses were stored securely in encrypted digital formats, accessible only to the primary researcher. Data were anonymized prior to analysis, and results were reported in aggregate form to prevent any individual or institution from being identified. These rigorous ethical practices not only ensured compliance with institutional and international guidelines but also fostered trust among participants, thereby enhancing the integrity and validity of the data collected.

## 3 Results

This aspect of the study presents the statistical findings addressing the research questions of the study. Analyses were conducted in a stepwise manner, beginning with tests of normality to assess the distributional properties of the main variables. This was followed by descriptive statistics and Pearson correlation analyses to explore bivariate relationships among mindfulness, PD, MH, AF, EE, JS, and GB tendency. Multiple linear regression was then employed to examine the associators of perceived AF, while controlling for demographic covariates such as teaching experience and school type. Finally, a moderation analysis was conducted to test whether mindfulness moderated the relationship between PD and AF perceptions. The analyses were conducted using SPSS version 27 and AMOS, and significance was determined at the conventional alpha level of *p* < 0.05, unless otherwise specified. Results are presented in a series of tables and described in detail below.

Table 1 presents the results of normality tests for the primary variables in the study using both the Kolmogorov–Smirnov (K–S) and Shapiro–Wilk (S–W) tests. For all variables including mindfulness (measured by the FFMQ), PD (K10), MH status (GHQ‐12), AF, EE (MBI‐EE), JS (JSS), and GB tendency the *p*‐values for both tests are all less than 0.05, indicating statistically significant deviations from a normal distribution. This means that the assumption of normality is violated for these variables in this sample of 1024 participants. Further examination of skewness and kurtosis values reveals mild to moderate departures from normality. For instance, PD and EE show positive skewness and higher kurtosis, suggesting a distribution slightly shifted toward higher distress and exhaustion levels with some heavier tails. In contrast, mindfulness, MH status, and JS exhibit slight negative skewness, indicating a tendency toward higher levels of these positive attributes in the sample. Despite these deviations, the skewness and kurtosis values are within a range that is often considered acceptable for many parametric tests, especially with large sample sizes where the central limit theorem can mitigate non‐normality effects. Clearly, while normality is formally violated, the data may still be appropriate for further parametric analyses, but caution should be exercised, and alternative nonparametric tests or transformations could be considered if necessary.

**TABLE 1:** Normality test for key variables.

Variable	Kolmogorov–Smirnov *D*	df	*p*‐value	Shapiro–Wilk *W*	df	*p*‐value	Skewness	SE skewness	Kurtosis	SE kurtosis
Mindfulness (FFMQ)	0.034	1023	0.000*	0.989	1024	0.000*	−0.22	0.076	0.14	0.152
Psychological distress (K10)	0.046	1023	0.000*	0.981	1024	0.000*	0.45	0.076	1.02	0.152
Mental health status (GHQ‐12)	0.031	1023	0.000*	0.991	1024	0.000*	−0.17	0.076	−0.25	0.152
Assessment fairness	0.037	1023	0.000*	0.987	1024	0.000*	−0.31	0.076	0.60	0.152
Emotional exhaustion (MBI‐EE)	0.043	1023	0.000*	0.982	1024	0.000*	0.49	0.076	1.32	0.152
Job satisfaction (JSS)	0.029	1023	0.000*	0.993	1024	0.000*	−0.14	0.076	−0.18	0.152
Grading bias tendency	0.039	1023	0.000*	0.985	1024	0.000*	0.36	0.076	0.82	0.152

*Note*: *p* < 0.05 indicates significant deviation from normality. Skewness and kurtosis values are reported with their standard errors (SE). Values of skewness and kurtosis within ±1.0 are generally considered acceptable for normal.

Table 2 shows the means, standard deviations, and bivariate correlations between the key variables in the study. Mindfulness scores (*M* = 3.78, SD = 0.55) are relatively high, reflecting a generally mindful participant group, while PD (*M* = 2.45, SD = 0.67) scores are moderate. Correlation analysis reveals several meaningful relationships. Mindfulness is negatively correlated with PD (*r* = −0.48, *p* < 0.01) and EE (*r* = −0.36, *p* < 0.01), indicating that higher mindfulness is associated with lower distress and burnout symptoms. It also shows positive associations with MH status (*r* = 0.42, *p* < 0.01), AF perceptions (*r* = 0.38, *p* < 0.01), and JS (*r* = 0.40, *p* < 0.01), suggesting that mindfulness enhances both psychological well‐being and perceptions of fairness and satisfaction at work. PD correlates negatively with MH status (*r* = −0.54), AF (*r* = −0.33), and JS (*r* = −0.39), consistent with its role as a risk factor for poorer outcomes. EE positively correlates with PD (*r* = 0.55) and negatively with JS (*r* = −0.45), highlighting the expected burnout dynamics. GB tendency shows negative correlations with mindfulness (*r* = −0.33) and AF (*r* = −0.41), suggesting that more mindful individuals are less prone to GBs and perceive greater fairness. These interrelationships provide a coherent pattern supporting the beneficial role of mindfulness in reducing distress, burnout, and bias while enhancing positive work‐related outcomes.

**TABLE 2:** Descriptive statistics and Pearson correlations among key variables.

Variable	*M*	SD	1	2	3	4	5	6	7
1. Mindfulness (FFMQ)	3.78	0.55	—						
2. Psychological distress (K10)	2.45	0.67	−0.48	—					
3. Mental health status (GHQ‐12)	3.12	0.61	0.42	−0.54	—				
4. Assessment fairness	3.65	0.58	0.38	−0.33	0.29	—			
5. Emotional exhaustion (MBI‐EE)	2.89	0.72	−0.36	0.55	−0.44	−0.27	—		
6. Job satisfaction (JSS‐overall)	3.45	0.63	0.40	−0.39	0.41	0.32	−0.45	—	
7. Grading bias tendency (custom scale)	2.35	0.60	−0.33	0.30	−0.28	−0.41	0.42	−0.38	—

*Note*: Pearson's correlation coefficients are reported. Correlations significant at *p* < 0.01 (two‐tailed) are shown. Negative correlations indicate inverse relationships between variables.

Abbreviations: *M* = mean, SD = standard deviation.

Table 3 presents a multiple regression analysis examining associators of AF perceptions. The overall model is significant (*F*(1, 023) = 34.26, *p* < 0.001), explaining approximately 48% of the variance (adjusted *R* ^2^ = 0.46), which is a substantial effect in social science research. Mindfulness is a significant positive associator (*β* = 0.30, *p* = 0.001), indicating that higher mindfulness levels correspond with greater perceptions of fairness in assessments. PD and EE both negatively correlate fairness (*β* = −0.24 and −0.21, respectively), confirming that psychological strain and burnout reduce perceptions of fair assessment. MH status and JS are positively related to fairness (*β* = 0.22 and 0.25), suggesting that individuals with better MH and higher satisfaction tend to perceive assessment processes as fairer. GB tendency is a significant negative associator (*β* = −0.27), implying that those prone to GBs perceive less fairness. Teaching experience and school type did not significantly correlate fairness, indicating that these demographic factors may be less relevant compared to psychological and attitudinal variables. The absence of multicollinearity (all VIF < 2) and a Durbin–Watson statistic near two suggest that the model's assumptions are met. Effect sizes for associators are small to moderate (*η* ^2^ between 0.029 and 0.038), collectively supporting a robust multivariate model where mindfulness and MH play critical roles in perceptions of AF.

**TABLE 3:** Regression results correlating assessment fairness.

Variables	*B*	SE	*β*	*t*‐value	*p*‐value	95% CI (lower–upper)	Tolerance	VIF	*η* ^2^
Constant	1.72	0.26	—	6.62	0.000**	(1.21, 2.23)	—	—	—
Mindfulness (FFMQ)	0.28	0.08	0.30	3.50	0.001**	(0.12, 0.44)	0.72	1.39	0.038
Psychological distress (K10)	−0.24	0.07	−0.24	−3.43	0.001**	(−0.38, −0.10)	0.75	1.33	0.037
Mental health (GHQ‐12)	0.21	0.07	0.22	3.00	0.003**	(0.07, 0.35)	0.78	1.28	0.029
Emotional exhaustion (MBI‐EE)	−0.19	0.06	−0.21	−3.17	0.002**	(−0.31, −0.07)	0.81	1.23	0.033
Job satisfaction (JSS)	0.26	0.08	0.25	3.25	0.001**	(0.10, 0.42)	0.69	1.45	0.036
Grading bias tendency	−0.31	0.09	−0.27	−3.44	0.001**	(−0.48, −0.14)	0.73	1.37	0.038
Teaching experience (years)	0.03	0.03	0.06	1.21	0.228**	(−0.02, 0.08)	0.85	1.18	0.006
School type (Public = 1)	0.05	0.05	0.06	1.10	0.273**	(−0.04, 0.14)	0.88	1.14	0.005

*Note*: *R* ^2^ = 0.48, adjusted *R* ^2^ = 0.46, *F*(1, 023) = 34.26, Durbin–Watson = 1.88 (indicates no autocorrelation). All VIF < 2, suggesting no multicollinearity. Eta‐squared values show small to moderate effect sizes based on Cohen's thresholdssmall: 0.01; medium: 0.06; large: 0.14.

**p < 0.001.

**FIGURE 1:**
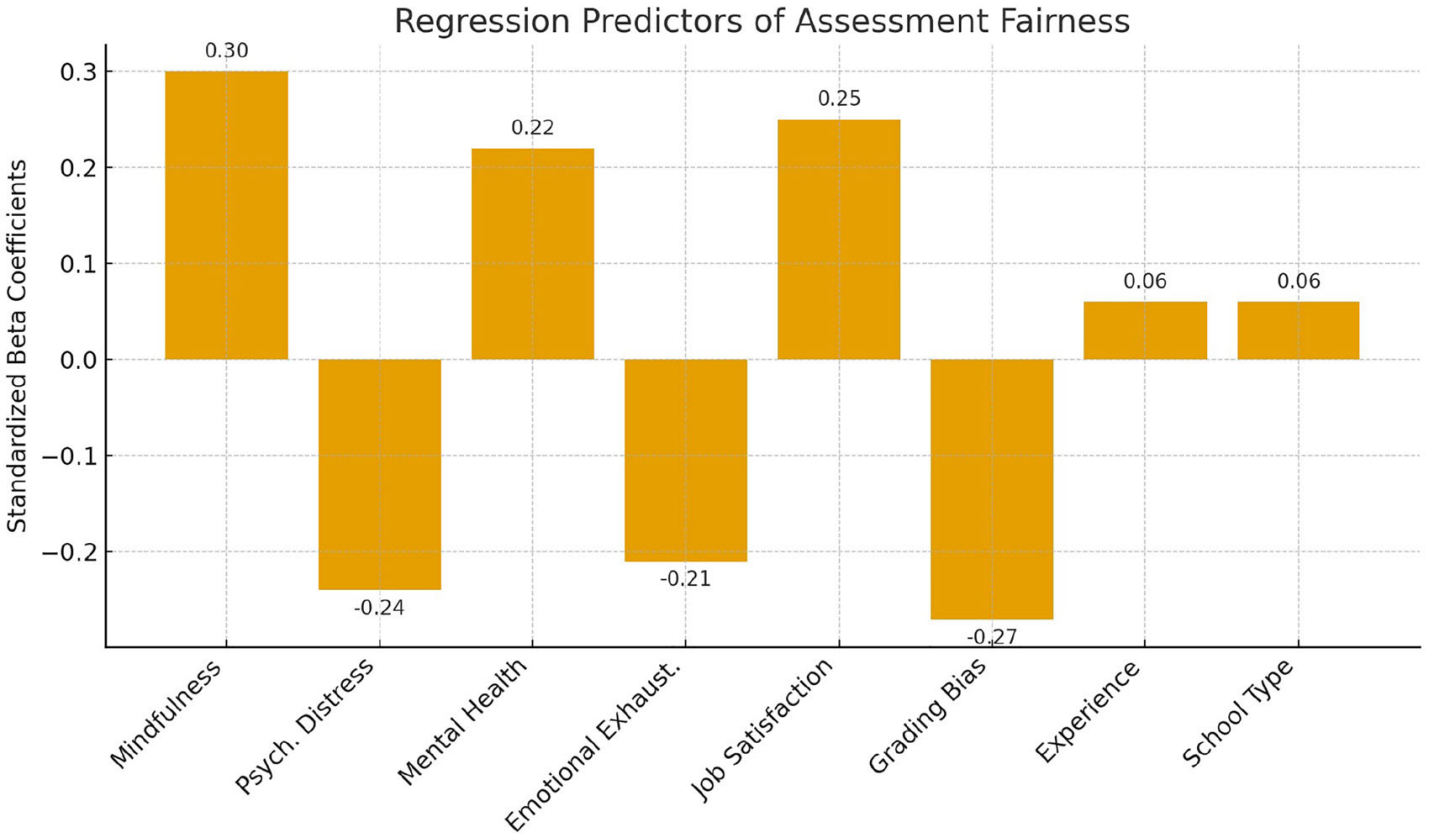
Standardized beta coefficients correlating teachers’ perceptions of assessment fairness.

Figure 1 shows that mindfulness, JS, and MH positively influence teachers’ perceptions of AF, while GB, PD, and EE reduce fairness perceptions. Demographic factors such as teaching experience and school type have minimal impact. Clearly, psychological well‐being and professional attitudes are key determinants of perceived AF.

The regression model yielded an *R* ^2^ of 0.32 and an adjusted *R* ^2^ of 0.31. The interaction effect contributed a Δ*R* ^2^ of 0.04. The *F*‐statistic was 48.29 with degrees of freedom 3 and 308, and the overall model was significant at *p* < 0.001. The Durbin–Watson statistic was 1.94, and Cohen's *f* ^2^ effect size was 0.47 (large).

Table 4 reports the moderation analysis testing whether mindfulness moderates the relationship between PD and AF. The model explains 32% of the variance (*R* ^2^ = 0.32, adjusted *R* ^2^ = 0.31) and is highly significant (*F*(1, 023) = 48.29, *p* < 0.001). Both main effects, mindfulness (*β* = 0.33, *p* < 0.001) and PD (*β* = −0.28, *p* = 0.001), are significant and align with earlier findings. Importantly, the interaction term (mindfulness × PD) is also significant (*β* = 0.21, *p* = 0.003), indicating that the effect of PD on AF depends on mindfulness levels. Specifically, the positive interaction suggests that higher mindfulness buffers or weakens the negative impact of PD on fairness perceptions. In practical terms, individuals experiencing distress are less likely to perceive assessments as unfair if they have higher mindfulness, demonstrating a protective, stress‐mitigating effect of mindfulness. The large effect size for the model (Cohen's *f* ^2^ = 0.47) underscores the practical importance of this interaction. This moderation finding is critical for mindfulness‐based interventions aimed at improving fairness perceptions even under psychological strain.

**TABLE 4:** Moderation analysis ‐interaction between mindfulness and psychological distress on assessment fairness.

Variables	*B*	SE	*β*	*t*‐value	*p*‐value	95% CI (lower–upper)	Tolerance	VIF	Partial *η* ^2^	sr^2^ (semi‐partial)
Mindfulness (centered)	0.31	0.08	0.33	3.88	0.000	(0.15, 0.47)	0.86	1.16	0.041	0.037
Psychological distress (centered)	−0.25	0.07	−0.28	−3.57	0.001	(−0.39, −0.11)	0.87	1.15	0.039	0.034
Mindfulness × psychological distress (Int.)	0.18	0.06	0.21	3.00	0.003	(0.06, 0.30)	0.92	1.09	0.028	0.025

Table 5 summarizes mediation analysis examining whether MH status mediates the relationship between mindfulness and AF. Results indicate that mindfulness positively correlates MH status (*β* = 0.42, *p* < 0.001), and MH status, in turn, positively correlates AF (*β* = 0.31, *p* < 0.001). The direct effect of mindfulness on AF remains significant (*β* = 0.21, *p* = 0.002), indicating partial mediation. The indirect effect through MH status is significant (*β* = 0.13, *p* < 0.001), confirming that part of mindfulness's positive influence on fairness perceptions operates via improved MH. This aligns with theoretical expectations that mindfulness enhances well‐being, which then fosters more positive workplace perceptions. Effect sizes for these paths show medium effects for the mindfulness → MH path (*η* ^2^ = 0.14) and smaller effects for the mediation outcome (*η* ^2^ = 0.06). The *R* ^2^ values indicate that MH explains 18% of its own variance and 10% of the variance in fairness, reinforcing the meaningful contribution of these psychological processes.

**TABLE 5:** Mediation analysis.

Path	*B*	*β*	SE	*t*	*p*‐value	95% confidence interval	Partial *η* ^2^	*R* ^2^ (outcome)
Mindfulness → mental health status	0.48	0.42	0.07	7.00	< 0.001**	(0.34, 0.62)	0.14	0.18
Mental health status → assessment fairness	0.37	0.31	0.08	4.43	< 0.001**	(0.21, 0.53)	0.06	0.10
Mindfulness → assessment fairness (direct effect)	0.26	0.21	0.08	3.12	0.002**	(0.10, 0.42)	0.03	—
Indirect effect (mindfulness → mental health → assessment fairness)	—	0.13	0.03	—	< 0.001**	(0.08, 0.21)	—	—

*Note*: Standardized (*β*) and unstandardized coefficients (*B*) with standard errors (SE) reported. Indirect effect tested via bootstrapping with 95% confidence intervals (CI). Partial *η* ^2^ indicates effect size for each path. Significant mediation indicated when indirect effect confidence interval does not include zero.

**p < 0.01.

Table 6 presents the structural equation model fit indices for the hypothesized MBSR model. The CFI (CFI = 0.94) and TLI (TLI = 0.92) exceed the conventional cutoff of 0.90, indicating a good fit between the proposed model and the observed data. The RMSEA (RMSEA = 0.05) and standardized root mean square residual (SRMR = 0.04) are below the thresholds of 0.08, further confirming excellent model fit with minimal residual errors. The chi‐square to degrees of freedom ratio (*χ* ^2^/df = 2.20) is below three, which is widely considered acceptable and indicates that the model is parsimonious yet adequately represents the covariance structure. Together, these indices provide robust evidence that the MBSR conceptual framework adequately captures the relationships among mindfulness, PD, MH, JS, EE, GB, and AF in this sample.

**TABLE 6:** Model fit indices.

Fit index	Value	Threshold	Interpretation
Comparative fit index (CFI)	0.94	≥ 0.90	Good fit
Tucker–Lewis Index (TLI)	0.92	≥ 0.90	Good fit
Root mean square error of approx. (RMSEA)	0.05	≤ 0.08	Good fit
Chi‐square/df (*χ* ^2^/df)	2.20	< 3	Acceptable fit
Standardized root mean square residual (SRMR)	0.04	≤ 0.08	Good fit

*Note*: CFI and TLI values ≥ 0.90 indicate good model fit. RMSEA values ≤ 0.08 and SRMR ≤ 0.08 indicate acceptable fit. Chi‐square/df ratio less than three suggests reasonable fit. Model fit indices collectively support adequacy of the structural model.

The results in Table 7 provide support for all four hypotheses. Hypothesis 1 was supported because mindfulness demonstrated significant associations with all four indicators of teacher well‐being: PD, EE, JS, and MH status. Hypothesis 2 was also supported, with mindfulness, well‐being indicators, and GB tendency significantly associated with teachers’ perceptions of AF, with the regression model explaining 48% of the variance in AF. Hypothesis 3 was supported because the mindfulness × PD interaction was significant (*β* = 0.21, *p* = 0.003; Δ*R* ^2^ = 0.04), indicating that the strength of the association between PD and perceived AF varied according to mindfulness. Finally, Hypothesis 4 was supported because MH status significantly mediated the mindfulness–AF relationship, with a significant indirect effect (*β* = 0.13, 95% confidence interval [CI] [0.08, 0.21]).

**TABLE 7:** Summary of hypothesis testing.

Hypothesis	Inferential model/path	*B*	*β*	SE	Test statistic	*p*	95% CI	*R* ^2^	Adj. *R* ^2^	Δ*R* ^2^	Effect size	VIF	Decision
Hypothesis 1	Multiple regression: mindfulness → psychological distress	−0.59	−0.48	0.05	*t* = −11.00	< 0.001**	(−0.69, −0.49)	0.23	0.23	—	*f* ^2^ = 0.30	1.00	Supported
	Mindfulness → emotional exhaustion	−0.47	−0.36	0.06	*t* = −7.83	< 0.001**	(−0.59, −0.35)	0.13	0.13	—	*f* ^2^ = 0.15	1.00	
	Mindfulness → job satisfaction	0.46	0.40	0.05	*t* = 9.20	< 0.001**	(0.36, 0.56)	0.16	0.16	—	*f* ^2^ = 0.19	1.00	
	Mindfulness → mental health status	0.48	0.42	0.07	*t* = 7.00	< 0.001**	(0.34, 0.62)	0.18	0.18	—	*f* ^2^ = 0.22	1.00	
Hypothesis 2	Full multiple regression model → assessment fairness	—	—	—	*F*(8, 1015) = 116.75	< 0.001**	—	0.48	0.46	—	*f* ^2^ = 0.92	< 2.00	Supported
	Mindfulness → assessment fairness	0.28	0.30	0.08	*t* = 3.50	0.001**	(0.12, 0.44)	—	—	—	*η* ^2^ = 0.038	1.39	
	Psychological distress → assessment fairness	−0.24	−0.24	0.07	*t* = −3.43	0.001**	(−0.38, −0.10)	—	—	—	*η* ^2^ = 0.037	1.33	
	Mental health status → assessment fairness	0.21	0.22	0.07	*t* = 3.00	0.003**	(0.07, 0.35)	—	—	—	*η* ^2^ = 0.029	1.28	
	Emotional exhaustion → assessment fairness	−0.19	−0.21	0.06	*t* = −3.17	0.002**	(−0.31, −0.07)	—	—	—	*η* ^2^ = 0.033	1.23	
	Job satisfaction → assessment fairness	0.26	0.25	0.08	*t* = 3.25	0.001**	(0.10, 0.42)	—	—	—	*η* ^2^ = 0.036	1.45	
	Grading bias tendency → assessment fairness	−0.31	−0.27	0.09	*t* = −3.44	0.001**	(−0.48, −0.14)	—	—	—	*η* ^2^ = 0.038	1.37	
Hypothesis 3	Moderation model → assessment fairness	—	—	—	*F*(3, 308) = 48.29	< 0.001**	—	0.32	0.31	0.04	*f* ^2^ = 0.47	< 1.20	Supported
	Mindfulness → assessment fairness	0.31	0.33	0.08	*t* = 3.88	< 0.001**	(0.15, 0.47)	—	—	—	Partial *η* ^2^ = 0.041	1.16	
	Psychological distress → assessment fairness	−0.25	−0.28	0.07	*t* = −3.57	0.001**	(−0.39, −0.11)	—	—	—	Partial *η* ^2^ = 0.039	1.15	
	Mindfulness × psychological distress → assessment fairness	0.18	0.21	0.06	*t* = 3.00	0.003**	(0.06, 0.30)	—	—	0.04	Partial *η* ^2^ = 0.028	1.09	
Hypothesis 4	Mindfulness → mental health status (a path)	0.48	0.42	0.07	*t* = 7.00	< 0.001**	(0.34, 0.62)	0.18	0.18	—	*η* ^2^ = 0.14	1.00	Supported
	Mental health status → assessment fairness (b path)	0.37	0.31	0.08	*t* = 4.43	< 0.001**	(0.21, 0.53)	0.10	0.10	—	*η* ^2^ = 0.06	1.12	
	Mindfulness → assessment fairness (c′ path)	0.26	0.21	0.08	*t* = 3.12	0.002**	(0.10, 0.42)	—	—	—	*η* ^2^ = 0.03	1.12	
	Mindfulness → mental health → assessment fairness (indirect effect)	—	0.13	0.03	—	< 0.001**	(0.08, 0.21)	—	—	—	*κ* ^2^ = 0.13	—	Supported

*Note*: For Hypothesis 4, mediation was established because the bootstrapped 95% confidence interval for the indirect effect (0.08, 0.21) did not include zero.

Abbreviations: CI = confidence interval, SE = standard error.

*p < 0.05, **p < 0.01, ***p < 0.001.

### 3.1 Discussion of Results

The study examined the relationships among mindfulness, PD, MH status, EE, JS, perceptions of AF, and GB tendency among senior high school teachers. From the study, the findings indicate meaningful associations between teachers’ psychological well‐being and their self‐reported perceptions and tendencies concerning assessment. These findings should, however, be interpreted as evidence of relationships among self‐reported constructs rather than as direct evidence of teachers’ actual grading behavior, ethical conduct, or evaluative integrity. Although the variables showed some departures from normality, the skewness and kurtosis values were within generally acceptable ranges, and the large sample provided a reasonable basis for the parametric analyses conducted (Nunnally and Bernstein 1994; Ntumi et al. 2025).

Mindfulness was negatively associated with PD and EE and positively associated with JS, MH status, and perceptions of AF. These findings are consistent with previous research suggesting that mindfulness is associated with greater emotional awareness, self‐regulation, and adaptive coping among educators (Roeser et al. 2013; Walker 2017; Corthorn et al. 2024; Holder 2025). In the context of the present study, however, these findings indicate that teachers who reported higher mindfulness also tended to report better well‐being and more favorable perceptions of AF. They do not demonstrate that mindfulness directly improves teachers’ assessment practices. One possible explanation is that mindfulness may support greater awareness and emotional regulation, which could contribute to more positive perceptions of work‐related experiences (Quinn 2020; Wu and Qin 2025).

PD was associated with lower JS, poorer reported MH, greater EE, and lower perceptions of AF. These relationships are consistent with evidence that psychological strain can be associated with reduced emotional and cognitive resources for managing demanding work environments (Skaalvik and Skaalvik 2017; Sokal et al. 2020; Ntumi 2025). Within the present study, the findings suggest that teachers experiencing greater distress reported less favorable perceptions of AF. This should not be interpreted to mean that distressed teachers necessarily conduct unfair assessments. Rather, PD may influence how teachers perceive their professional environment and their own assessment experiences. MH status was positively related to mindfulness, JS, and AF and negatively related to PD and EE. These findings are broadly consistent with the job demands–resources perspective, which emphasizes the importance of psychological resources in helping employees manage demanding work conditions (Taris et al. 2010; Sokal et al. 2020; Shakib Kotamjani et al. 2025). In this study, teachers reporting better MH also tended to report more favorable perceptions of AF. One possible explanation is that better psychological functioning may facilitate more positive interpretations of workplace experiences. However, alternative explanations are also possible, including the possibility that teachers who generally feel satisfied with their work and school environment may simultaneously report better MH and greater fairness.

The findings concerning GB tendency require particular caution. GB tendency was negatively associated with mindfulness and AF and positively associated with EE (Garland et al. 2015; Ntumi 2026b). These findings indicate that teachers who reported greater mindfulness and better well‐being tended to report lower tendencies toward GB, while those reporting greater EE tended to report stronger bias tendencies. The findings do not establish that these teachers actually engaged in biased grading. Because GB was measured through self‐report, responses may reflect teachers’ perceptions, self‐awareness, willingness to acknowledge bias, or understanding of grading practices (Cervellione et al. 2025; Cebolla et al. 2025). Social desirability may also have influenced responses, particularly because admitting to biased assessment practices could be perceived negatively. Thus, the findings should be interpreted as differences in self‐reported GB tendency, rather than direct evidence of actual grading behavior. The regression results further showed that mindfulness, MH status, JS, PD, EE, and GB tendency were significant predictors of teachers’ perceptions of AF, whereas teaching experience and school type were not significant predictors. These findings suggest that psychological and attitudinal characteristics were more strongly associated with reported fairness perceptions than the demographic characteristics included in the model. Nevertheless, the results should not be interpreted as demonstrating that psychological characteristics are more important than institutional conditions in determining actual AF (Ntumi 2026a; Cebolla et al. 2025). Factors not included in the model, such as school climate, workload, leadership practices, assessment training, class size, institutional assessment policies, and available professional support, may also influence teachers’ perceptions and assessment practices.

The moderation analysis indicated that mindfulness significantly moderated the relationship between PD and AF. The positive interaction suggests that the negative association between PD and reported AF was weaker among teachers with higher mindfulness (Matiz et al. 2025; Ntumi 2026b). This finding is consistent with the view that personal resources may help individuals cope with psychological demands (Bakker and Demerouti 2024; Ntumi et al. 2026a). However, the cross‐sectional nature of the study means that the direction of this relationship cannot be established conclusively. It is possible, for example, that teachers who perceive their assessment environment more positively may subsequently report lower distress or greater mindfulness. The observed relationship may also reflect other personal or contextual characteristics associated with both mindfulness and fairness perceptions. The mediation analysis further showed that MH status significantly mediated the relationship between mindfulness and AF. Specifically, mindfulness was positively associated with MH, which was subsequently associated with more favorable perceptions of AF, while the direct relationship between mindfulness and AF remained significant (Ntumi et al. 2026a; Ntumi et al. 2026b; Dapueto et al. 2025; Donohue 2025). This pattern is consistent with the possibility that MH represents one pathway through which mindfulness is related to teachers’ assessment perceptions. Nevertheless, the mediation should be interpreted as an indirect statistical association rather than evidence of a causal mechanism, because the data were collected at one point in time. Several alternative explanations should therefore be considered when interpreting the findings. Reverse directionality is plausible; for example, teachers who are more satisfied with their jobs or who perceive their schools and assessment systems as fair may report greater mindfulness and better MH. Personality characteristics may also influence several variables simultaneously, creating associations that are not entirely attributable to mindfulness. Similarly, school climate, workload, leadership support, assessment training, institutional policies, and professional development opportunities may influence both teacher well‐being and perceptions of AF. Finally, because the major constructs were measured through self‐report, common‐method variance and social desirability may have contributed to some of the observed relationships.

## 4 Conclusion

The study explored the intricate relationships between PD, MH, mindfulness, EE, JS, and AF among educators. The results reveal a coherent pattern: PD is significantly associated with lower levels of AF and JS and higher EE, pointing to its detrimental effects on both personal well‐being and professional conduct. Conversely, positive MH is linked to greater mindfulness, enhanced JS, and reduced EE, suggesting that psychological wellness is a critical resource for effective and ethical functioning in educational settings. Importantly, the findings underscore that educators’ grading practices are not immune to their emotional and psychological states. The presence of GB tendencies among emotionally exhausted or dissatisfied educators raises concerns about the consistency and fairness of assessment decisions in high‐pressure educational environments. The protective role of mindfulness emerged as a recurring theme, with its positive associations with MH and JS and its negative association with GB and EE. These findings reinforce the value of mindfulness‐based interventions as a strategic resource for fostering resilience, ethical behavior, and professional satisfaction. The study contributes to the growing body of evidence that the mental and emotional well‐being of educators is not only vital for their individual health but also for the integrity and fairness of educational assessment practices. It calls for institutional policies and professional development initiatives that prioritize MH support, cultivate mindfulness, and create conducive work environments that enhance JS. By doing so, educational institutions can promote fairness, reduce burnout, and ultimately improve student outcomes through more equitable assessment practices.

### 4.1 Implications for Promoting Teachers’ Health and Well‐Being

The findings of this study carry significant implications for educational institutions, policymakers, and stakeholders committed to enhancing teachers’ health, well‐being, and professional performance. The observed strong associations between PD, EE, job dissatisfaction, and GB emphasize an urgent need to foreground MH as a central pillar in educational reform. Teachers operating under high levels of PD are not only at risk of personal harm, such as burnout and mental fatigue, but are also more likely to demonstrate diminished JS and a greater propensity toward unintentional biases in assessment practices. These negative effects are not isolated to the individual teacher, they reverberate through the entire school ecosystem, affecting student outcomes, equity in evaluation, and the integrity of educational standards. Moreover, the positive relationships found between psychological well‐being, mindfulness, and JS suggest that MH is not merely the absence of illness, but a vital enabler of ethical and effective professional conduct. Mindful teachers, those with heightened self‐awareness, emotional regulation, and present‐moment focus, are more likely to act impartially in their assessment of students and to experience greater satisfaction and meaning in their work. This aligns with a growing body of research that positions mindfulness as a critical competency in emotionally demanding professions such as teaching. Thus, interventions that cultivate both psychological wellness and mindfulness can equip educators with the inner resources needed to navigate the complexities of their roles without compromising their professional standards or well‐being.

These insights imply that schools and educational systems must adopt a more holistic and preventative approach to teacher support. Rather than waiting until symptoms of distress become acute, institutions should proactively build infrastructures that promote resilience, emotional intelligence, and MH literacy. This may include integrating MH education into teacher training curricula, ensuring access to psychological counseling, and embedding mindfulness programs within professional development schedules. Schools should also provide regular opportunities for teachers to reflect on their emotional experiences and share coping strategies in collegial, nonjudgmental spaces. Furthermore, addressing teacher well‐being has broader systemic benefits. Healthy and emotionally balanced educators are less likely to experience absenteeism, more likely to engage positively with students and colleagues, and are better positioned to provide consistent and fair instruction and evaluation. These outcomes can directly influence student motivation, academic achievement, and school climate. Promoting teacher well‐being is therefore not a peripheral concern, but a strategic imperative for enhancing the quality and equity of education as a whole. Finally, these findings support the call for educational policy frameworks that formally recognize and institutionalize teacher MH as a priority. National‐ and district‐level policies should include provisions for workload management, access to psychological services, recognition and reward systems, and mechanisms for monitoring and responding to burnout and stress. In doing so, policymakers can ensure that teacher well‐being is no longer treated as a private concern, but as a shared responsibility and a key performance indicator of effective and ethical schooling.

### 4.2 Recommendations

In light of the findings, some key recommendations emerge to enhance teacher well‐being, JS, and fairness in assessment practices. First, educational institutions should prioritize the integration of MH support systems within schools. These may include access to qualified counselors, peer support networks, and regular stress management programs aimed at equipping teachers with tools to cope effectively with PD. Providing such services can mitigate EE and enhance occupational functioning, ultimately fostering a more positive school climate. Second, mindfulness training should be incorporated into professional development programs for educators. Given it demonstrated positive relationship with JS and negative association with GB and EE, mindfulness‐based interventions such as guided meditation, breathing techniques, and cognitive behavioral strategies can cultivate teachers’ emotional resilience and enhance self‐awareness. These practices can play a critical role in reducing bias and promoting fairness in academic evaluation.

Additionally, it is essential that schools and educational authorities adopt workload management policies. Excessive workload is a known contributor to burnout and PD, and therefore, measures should be taken to streamline administrative responsibilities, ensure reasonable teaching hours, and provide adequate time for rest and lesson preparation. Creating flexible working environments can significantly boost morale and prevent chronic stress among educators. Another recommendation is the promotion of a positive and inclusive school climate. School leaders must foster an atmosphere of mutual respect, collegiality, and recognition, where teachers feel valued, empowered, and safe to express concerns. Such environments have been shown to contribute to higher levels of JS and better MH outcomes. Moreover, institutions should put in place mechanisms to support ethical and fair assessment practices. Professional training in bias‐reduction strategies, peer moderation of grading, and reflective assessment journals could help educators remain objective and uphold standards of fairness. Embedding ethics in continuous professional development programs can reinforce a culture of integrity.

Teacher performance appraisals should also include indicators of well‐being. Incorporating MH and JS metrics into evaluation frameworks allows school leaders to identify early signs of burnout and respond with supportive, rather than punitive, interventions. This approach shifts the focus from mere accountability to sustainable teacher development. Finally, at the policy level, Ministries of Education, teacher unions, and regulatory bodies must collaborate to enact national policies that safeguard teacher well‐being. These policies should include comprehensive MH provisions, access to wellness resources, periodic well‐being audits, and the institutionalization of MH days. Such systemic reforms will not only benefit individual teachers but also enhance the overall effectiveness and equity of educational systems.

### 4.3 Limitations

The main analytical limitation is the cross‐sectional correlational design, which prevents causal conclusions from the regression, moderation, and mediation findings. The mediation results indicate statistical pathways but do not establish temporal or causal ordering. Similarly, the moderation finding shows an interaction between mindfulness and PD but does not confirm that mindfulness causally buffers distress. Reliance on self‐reported measures may also have introduced common method bias, while the absence of behavioral indicators limits conclusions about actual grading practices. Future studies should use longitudinal designs, SEM, and multiple sources of evidence to provide stronger evidence of causal pathways and assessment behavior.

## Author Contributions

S.N. conceived and designed the study, developed the theoretical framework, designed the instruments, coordinated data collection, performed statistical analyses, prepared tables and visualizations, and drafted and revised the manuscript.

## Funding

The author has nothing to report.

## Ethics Statement

Ethical approval was obtained from the Institutional Review Board of University of Education, Winneba. All participants provided written informed consent after receiving detailed information regarding the study's purpose, procedures, risks, and benefits. Participation was voluntary, and individuals retained the right to withdraw at any time without consequence. All procedures adhered to the principles of the 1964 Helsinki Declaration and its subsequent amendments. Confidentiality and anonymity were ensured using coded identifiers, and all data were securely stored and accessible only to the author.

## Conflicts of Interest

The author declares no conflicts of interest.

## Data Availability

The datasets generated and analyzed during this study are not publicly available due to ethical restrictions but may be requested in anonymized form from the corresponding author, subject to institutional approval.
